# A case of recurrent hematuria in primary prostatic low grade mucosa associated lymphoid tissue 

**DOI:** 10.15171/jnp.2017.08

**Published:** 2016-10-22

**Authors:** Saeed Hashemzadeh, Farid Farrokhi, Amir Hozhabrossadaty, Kamran Ghafarzadegan, Hami Ashraf

**Affiliations:** ^1^Razavi Cancer Research Center, Research and Education Department, Razavi Hospital, Mashhad, Iran; ^2^Pathology Ward, Research and Education Department, Razavi Hospital, Mashhad, Iran; ^3^Urology Ward, Research and Education Department, Razavi Hospital, Mashhad, Iran

**Keywords:** Recurrent hematuria, Immunohistochemistry, Mucosa-associated lymphoid tissue, Lymphoma, Prostate

## Abstract

**Background:**

Primary mucosa-associated lymphoid tissue (MALT) lymphoma is a rare malignancy. We found only 8 cases of MALT lymphoma in literature.

**Case Presentation:**

We report here another case of primary prostatic MALT lymphoma which is presented by hematuria and diagnosed primarily as BPH. Immunohistochemistry studies demonstrate the diagnosis and MALT lymphoma. Six months after starting the treatment the patient was alive and well.

**Conclusions:**

Prostatic MALTomas are mainly presented with urinary obstruction or hematuria and have an indolent growth with a good prognosis.

**Implication for health policy/practice/research/medical education:**

Although primary mucosa-associated lymphoid tissues (MALTs) of the prostate are very rare tumors, it could be considered as a potential pathology underlying of unjustified urinary obstruction, or hematuria..

## 1. Introduction

Prostate lymphomas and especially primary mucosa-associated lymphoid tissue (MALT) lymphoma is a rare malignancy. To date, only 8 cases of primary MALT lymphoma are reported all over the world. The first case of MALT lymphoma of the prostate was reported in 1992 by Franco et al (1) and the last one is a case from Japan which is reported by Koga et al in 2009 (2). We report here another case of primary prostatic MALT lymphoma which is presented by hematuria and diagnosed primarily as benign prostatic hyperplasia (BPH). It should also be noted that the present patient is a case of four transfusions including four units of packed cells (PC), five units of fresh frozen plasma (FFP), and 3 units of whole blood.

## 2. Case Presentation

A 63-year-old man presented with hematuria and urinary obstruction symptoms. He came to the hospital four times each with presence of gross hematuria. Moreover, the patient suffered from frequency and nocturia at least for six months. Physical examination revealed enlargement of prostate, but there was no palpable nodule on the digital rectal examination. PSA was normal and there was also no evidence of hepatomegaly or splenomegaly.

The first transurethral resection of the prostate (TURP) and biopsy was done for him, but pathologic results showed the evidence of BPH. After 28 days, the patient was admitted again due to gross hematuria. Rectal examination was normal. Pelvic CT-scan revealed a big clot in bladder without any lymphadenopathy. There was a significant heterogeneity in prostate. In second TURP, clot evacuation and biopsy were performed on more than 15 different areas of prostate. During the next 2 weeks, the patient underwent 3 other trans-urethral coagulation and clot evacuations due to hematuria.

**Figure 1:**
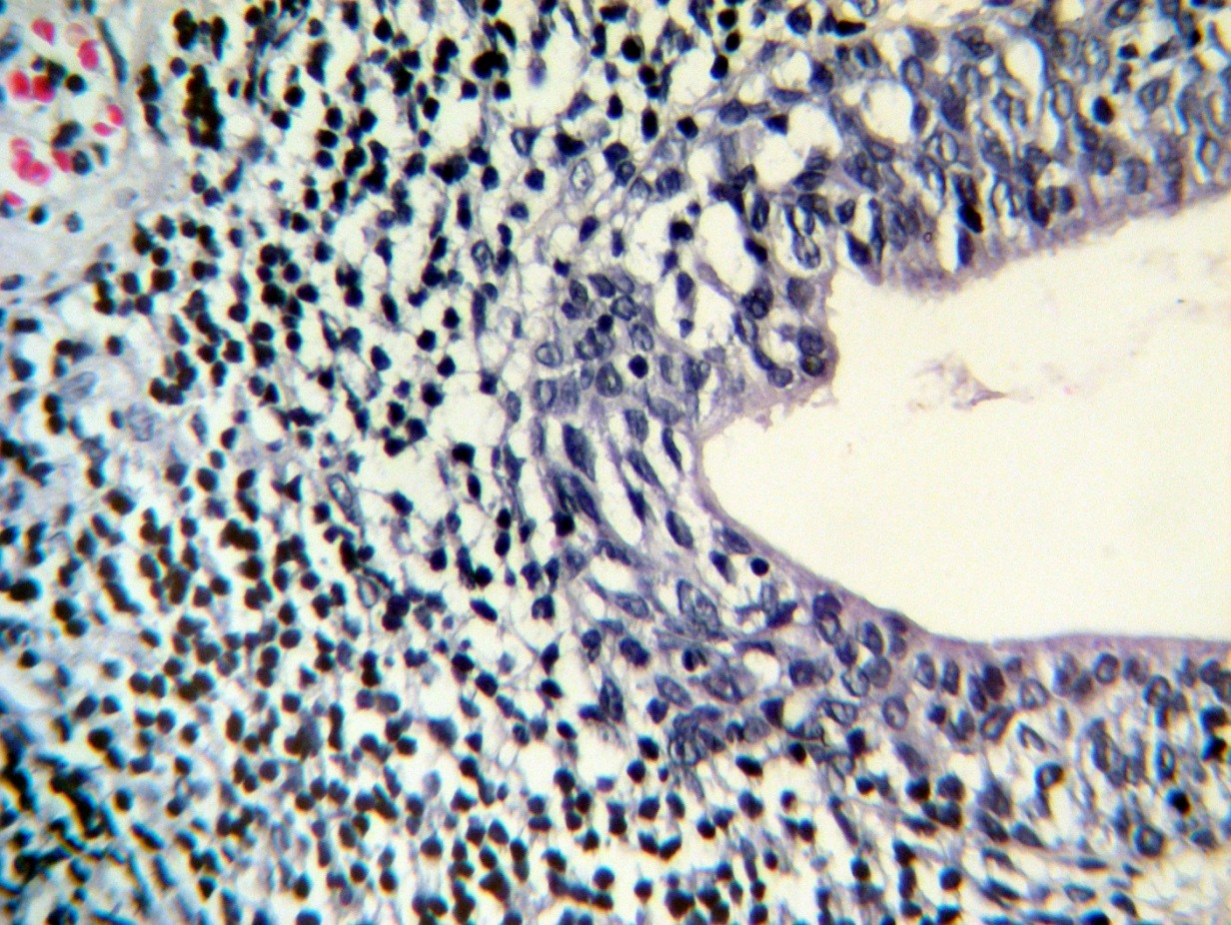
Neoplastic lymphoid and invasion to the large ductal epithelium (x400).

**Figure 2:**
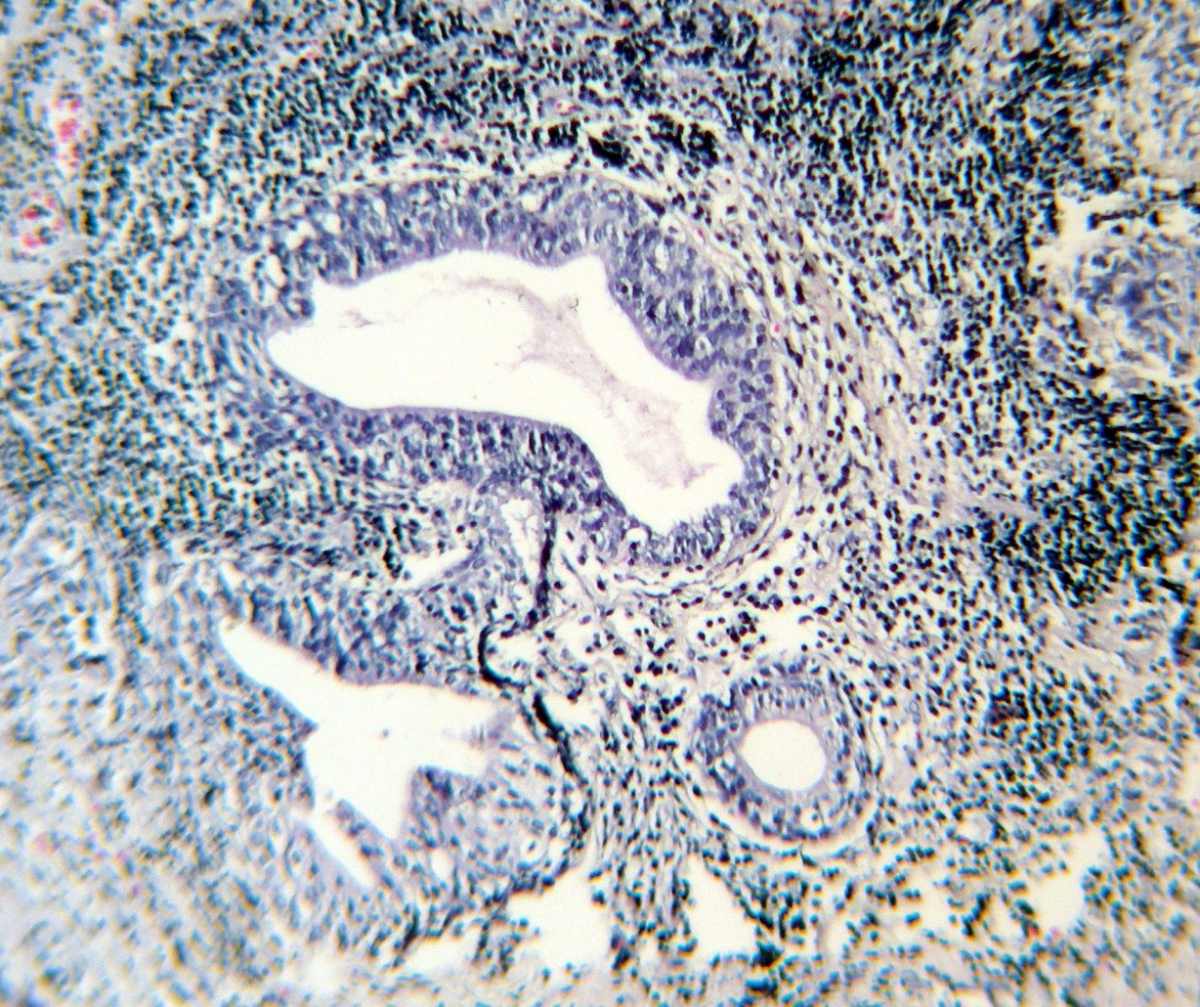
Monomorphic neoplastic lymphoid cells with hyperchromatic nuclei (x400).

However, laboratory tests such as prothrombin time (PT) , partial thromboplastin time (PTT), clotting time (CT), bleeding time (BT) and platelet count were normal. After 15 days hematuria was stopped and was not repeated. Sections from prostate show foci of hemorrhage, Figures 1 and 2).

The first transfusion was 2 units of PC during the second surgery. The second transfusion was 2 units of PC and 2 FFP, two days later. The consecutive third and fourth transfusions were done two days later during which the patient received 3 units of FFP, and 3 units of whole blood.

**Figure 3:**
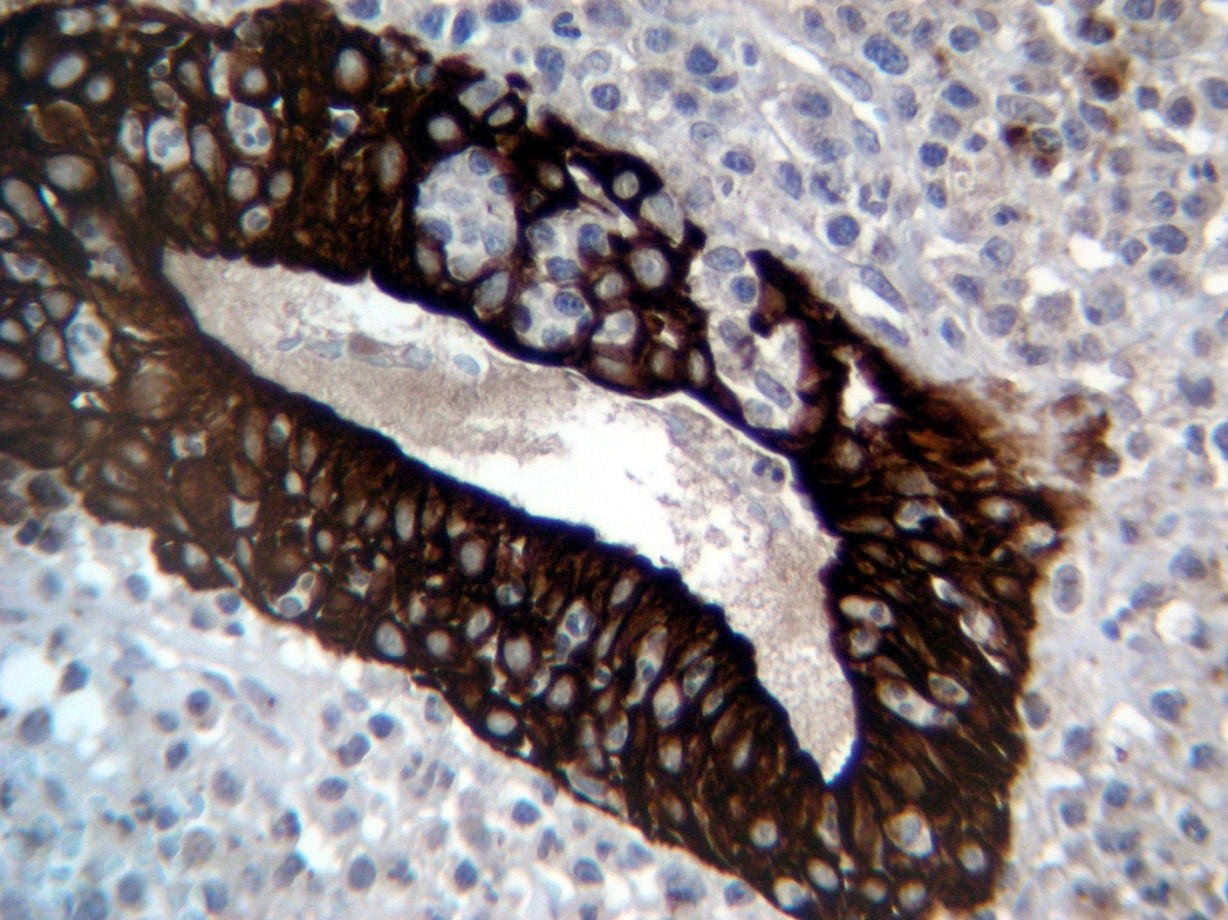
Immunohistochemical staining shows CD20-Positive lymphoid cells (x400).

Immunohistochemistry studies demonstrate CD20-positive in 90% of lymphoid cells and in the lymphoepithelial lesions (Figure 3), CD5-positive in background lymphocytes, CD43-positive in 90% of lymphoid cells, CD3-positive in background lymphocytes, CK and PSA markers are negative in neoplastic cells. Further evaluation and examination such as bone marrow biopsy, abdominal and pelvic CT-scan did not show other involvement.

In the last fallow up, around eight months after discharging, the patient was alive and asymptomatic. Moreover, there is no evidence of other organ involvement.

## 3. Discussion

According to the Revised European-American Lymphoma (REAL) classification MALT lymphomas were categorized as extra nodal marginal zone lymphomas of MALT-type. These groups of lymphomas are composed of mature B cells that are CD20, CD21, CD35 and IgM positive, and CD5, CD10 and IgD negative. Low-grade marginal zone lymphomas are composed of multifocal proliferations of polymorphous lymphoid cells including small lymphocytes with dark round nuclei, small lymphocytes with irregular nuclear contours and pale cytoplasm. For distinguishing reactive follicles from follicles invaded by lymphoma, immunhistochemical determination of the number and distribution of CD20-positive B cells and CD3-positive T cells may be useful (10).

The stomach is the most common site for MALT lymphomas (9). Histologic studies have showed that more than 90% of lymphomas are associated with *Helicobacter pylori* gastritis. The frequency of primary prostatic lymphoma is about 0.1% of non-Hodgkin’s lymphomas and 0.09% of all prostatic malignancies (3). Some of MALT lymphomas are observed in the breast, bladder, conjunctiva, kidney, liver, lung, skin, salivary glands, thyroid and thymus. However, these are not usual sites for MALT lymphomas (8). There is often a history of chronic inflammatory disorder or autoimmune disease such as Sjogren’s syndromes, Hashimoto’s thyroiditis and so on (3). Primary MALT lymphoma of prostate is a rare disease. Koga et al have impressively summarized the previous eight cases of MALT lymphoma. The comparison of these nine patients is shown in Table 1. Our case is younger than the mean age of 73 years which is mentioned in Koga and colleagues report, but the presentation symptoms were urinary obstruction as the same (2).

**Table 1:** The comparison of nine cases of MALT lymphoma

** Case**	** Age (y) **	** Symptom **	** Laboratory findings **	** Clinical stage **	** Treatment**	** Response **	**Outcome**	** Follow-up (mon) **	**Ref.**
1	75	Hematuria, pyuria	Normal	II	TUR, Cx	CR	AW	12	1
2	57	Urinary obstruction	Normal	I	TUR, Cx	CR	AW	18	3
3	84	Urinary obstruction	Elevated PSA	I	TUR	CR	DOC	24	4
4	67	Prostatism	Normal	I	TUR, Rx	CR	AW	36	5
5	87	Urinary obstruction	Normal	I	TUR	NR	NR	NR	6
6	79	Urinary obstruction	Normal	I	TUR	CR	AW	108*	7
7	70	Disuria	Elevated PSA	I	TUR, Cx	CR	AW	5	8
8	67	Urinary obstruction	Elevated PSA	I	Rx	CR	AW	15	2
9	57	dysuria, nocturia, residua	Elevated PSA	I	Rx	CR	AW	24	9
10	48	Urinary obstruction	Normal	I	Rx	CR	AW	6	10
11	63	Urinary obstruction, hematuria	Normal	I	TUR	CR	AW	6	Present case

## 4. Conclusions

As mentioned before these kinds of tumors are very rare and the 10 previously reported cases in the literature are not enough to certainly discuss about the best treatment option and prognosis. But it seems that these prostatic MALTomas have an indolent growth with a good prognosis (8,10). According to the published reports, prostatic MALTomas are mainly presented with urinary obstruction, or hematuria. Chemotherapy, transurethral resection, and external radiotherapy are successfully applied treatment options (1-10).

## Authors’ contribution

SH; study design, preparation of manuscript, final approval. FF; study design, data gathering, and final revision. AH and KG; study design, manuscript edition, final approval. HA; study design, data interpretation and manuscript preparation and final approval.

## Conflicts of interest

The authors declared no competing interests.

## Funding/Support

No special source of funding.
